# Using citation tracking for systematic literature searching - study protocol for a scoping review of methodological studies and an expert survey

**DOI:** 10.12688/f1000research.27337.1

**Published:** 2020-12-01

**Authors:** Julian Hirt, Thomas Nordhausen, Christian Appenzeller-Herzog, Hannah Ewald

**Affiliations:** 1 Institute of Applied Nursing Science, Department of Health, Eastern Switzerland University of Applied Sciences (formerly FHS St.Gallen), St.Gallen, Switzerland; 2 International Graduate Academy, Institute of Health and Nursing Science, Medical Faculty, Martin Luther University Halle-Wittenberg, Halle (Saale), Germany; 3 University Medical Library, University of Basel, Basel, Switzerland

**Keywords:** Citation Tracking, Literature Search, Supplementary Search, Methods, Scoping Review, Research Methodology, Survey, Systematic Review

## Abstract

**Background:** Up-to-date guidance on comprehensive study identification for systematic reviews is crucial. According to current recommendations, systematic searching should combine electronic database searching with supplementary search methods. One such supplementary search method is citation tracking. It aims at collecting directly and/or indirectly cited and citing references from "seed references”. Tailored and evidence-guided recommendations concerning the use of citation tracking are strongly needed.

**Objective:** We intend to develop recommendations for the use of citation tracking in health-related systematic literature searching. Our study will be guided by the following research questions: What are the benefits of citation tracking for health-related systematic literature searching? Which perspectives and experiences do experts in the field of literature retrieval methods have with regard to citation tracking in health-related systematic literature searching?

**Methods:** Our study will have two parts: a scoping review and an expert survey. The scoping review aims at identifying methodological studies on benefits or problems of citation tracking in health-related systematic literature searching with no restrictions on study design, language, and publication date. We will perform database searching in MEDLINE, The Cumulative Index to Nursing and Allied Health Literature (CINAHL), Web of Science Core Collection, two information science databases, and free web searching. Two reviewers will independently assess full texts of selected abstracts. We will conduct direct backward and forward citation tracking on included articles. The results of the scoping review will inform our expert survey through which we aim to learn about experts΄ perspectives and experiences. We will narratively synthesize the results and derive recommendations for performing health-related systematic reviews.

## Introduction

Systematic reviews are considered to be of high clinical and methodological importance as they help to derive recommendations for health care practice and future research ^1– 3^. A comprehensive literature search aiming to identify the available evidence as completely as possible is the foundation of every systematic review ^4– 6^. Due to an ever-growing research volume, lack of universal taxonomy and indexation, as well as extensive time requirements for identifying studies in a systematic way, effective search approaches are required ^5, 7, 8^. According to current recommendations, systematic search approaches should include both electronic database searching and one or several supplementary search methods ^9^. Potential supplementary search methods include citation tracking, contacting study authors or experts, handsearching, trial register searching, and web searching ^10^. In this study, we focus on citation tracking.

**Figure 1.**
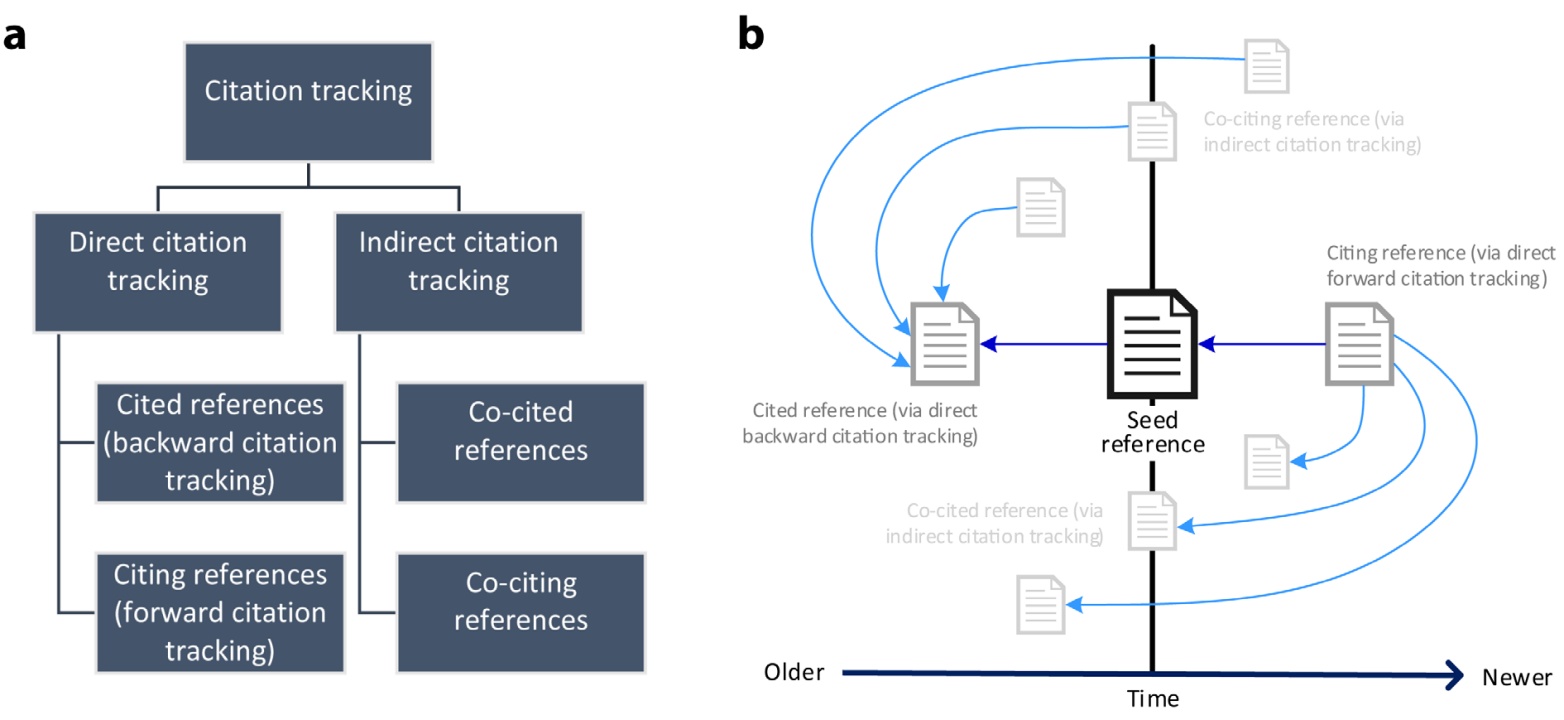
Overview of citation tracking methods. **1a**: Hierarchical illustration of different citation tracking methods; **1b**: Direct and indirect citation relationships of references based on a seed reference. A → B denotes A cites B.

Citation tracking is an umbrella term for multiple methods which directly or indirectly collect related references from so called "seed references". These references are usually eligible for inclusion into the review and known at the start of the citation search ^10– 12^. In the workflow of a systematic review, seed references for citation tracking often are records derived from screening bibliographic database search results. The taxonomy used to describe the principles of citation tracking is non-uniform and heterogeneous ^13– 16^. Citation tracking methods are sub-categorized into *direct* and *indirect* citation tracking ( Figure 1a). For direct citation tracking, the words "backward" and "forward" denote the directionality of tracking ^13, 17, 18^. Backward citation tracking is the oldest form of citation tracking. It aims at identifying references cited by a seed reference - which can easily be achieved by checking the reference list. Terms like "footnote chasing" or "reference list searching" are synonyms ^6, 13^. In contrast, forward citation tracking or chaining aims at identifying citing references, i.e. references that cite a seed reference ^19^. *Indirect* citation tracking describes the identification of (i) co-cited references or co-citations (i.e. other references cited by citing literature of a seed reference) and of (ii) co-citing references (i.e. references sharing references with a seed reference) ^11, 20^. Direct and indirect citation relationships of references based on a seed reference are illustrated in Figure 1b. Both direct and indirect citation tracking may contain one or more layers of iteration. To this end, researchers may use newly retrieved references as new seed references.

Direct backward citation tracking of cited references is currently the most common citation tracking method. However, recent guidance suggests that combining several citation tracking methods (e.g. screening cited, citing, co-cited and co-citing references) may be the most effective way to use citation tracking for systematic reviewing. It is quite possible that the added value of any form of citation tracking is not the same for all systematic reviews. It rather depends on a variety of factors. For instance, citation tracking may be especially beneficial in case of (i) complex searches (e.g. for reviews on public health topics), (ii) searching for health outcome measurement instruments, or (iii) research areas without consistent taxonomy, with vocabulary overlaps with other fields, or with a lack of index terms in databases (e.g. methodological topics) ^10, 20, 21^. Hence, tailored and evidence-guided recommendations on the use of citation tracking are strongly needed. However, none of the current topical reviews has in fact systematically identified available evidence on the use and benefits of citation tracking in the context of systematic literature searching ^10^.

Therefore, the aim of our study is to develop recommendations for the use of citation tracking in health-related systematic literature searching and to identify research gaps. Our study will be guided by the following two research questions:

- What are the benefits of citation tracking for health-related systematic literature searching?
- Which perspectives and experiences do experts have with regard to citation tracking in health-related systematic literature searching?

## Protocol

This protocol is reported according to the “Preferred Reporting Items for Systematic review and Meta-Analysis Protocols” (PRISMA-P) checklist ^22^. Details on the structure of our manuscript reporting related to PRISMA-P are provided elsewhere ^23^. Our study will have two parts: a scoping review and an expert survey. The scoping review has the objective to map the benefits or problems of citation tracking or, if that is not sufficiently informative, possible gaps in related research. The objective of the expert survey is to identify the perspectives, experiences and perceptions of experts in the field of literature retrieval methods with regard to citation tracking. Together, the scoping review and expert survey will serve as a basis to derive implications and recommendations for current practice and future research ^24– 26^. For the scoping review, we will use the framework by Arksey and O’Malley ^26, 27^ and the “Preferred Reporting Items for Systematic reviews and Meta-Analyses extension for Scoping Reviews” (PRISMA-ScR) ^28^. Since an expert survey can be part of a scoping review, and the scoping review and the expert survey serve the same overall aim, we will follow methodological recommendations for scoping reviews ^25, 26^ and the “Strengthening The Reporting of Observational Studies in Epidemiology” (STROBE) statement ^29^.

### Scoping review

***Eligibility criteria.*** We will include any study with a focus on citation tracking as a means of evidence retrieval method which exhibits one of the following criteria: benefits/problems and/or effectiveness of (i) citation tracking in general; (ii) different methods of citation tracking (e.g. backward vs. forward, direct vs. indirect); or (iii) technical uses of citation tracking (e.g. comparing platforms and/or tools, e.g. Scopus vs. Web of Science, Oyster, Voyster). Eligible studies need to have a health-related context. Studies without an explicitly specified research context are also eligible. There will be no restrictions on study design, language, and publication date.

We will exclude studies solely using citation tracking for evidence retrieval, e.g. a systematic review applying citation tracking as a supplementary search technique, or studies focussing on citation tracking as a means to explore network or citation impact (i.e. bibliometric analysis). Additionally, we will exclude methodological guidelines only mentioning or recommending citation tracking without describing or assessing it. Furthermore, we will exclude editorials, commentaries, letters and abstract-only publications. Table 1 illustrates our inclusion and exclusion criteria per domain.

**Table 1.** Inclusion and exclusion criteria.

Domain	Inclusion criteria	Exclusion criteria
Study focus	Any study with a focus on citation tracking as an evidence retrieval method AND one of the following criteria: - any study assessing benefits/problems and/or effectiveness of citation tracking - any study comparing different methods of citation tracking (e.g. backward vs. forward, direct vs. indirect) - any study assessing technical uses of citation tracking (e.g. comparing platforms and/or tools, e.g. Scopus vs. WoS, Oyster, Voyster, etc.)	Any study solely using citation tracking for evidence retrieval (e.g. a systematic review applying citation tracking as supplementary search technique) OR any study solely assessing benefits and/or use and/or effectiveness of citation tracking to explore a network or citation impact (i.e. bibliometric analysis) OR any study describing solely the method of citation tracking without further describing or assessing it (focus citation tracking, search methods, systematic reviews or other types of reviews), e.g. guidelines for developing search strategies or guidelines for systematic or other reviews
Research context	Health-related or not specified	Other research contexts
Language	All languages	-
Publication year	All publication years	-
Publication type	Any	Editorials Commentaries Letters Abstract-only publications

***Information sources.*** We will search MEDLINE via Ovid, CINAHL (Cumulative Index to Nursing and Allied Health Literature), LLISFT (Library Literature & Information Science Full Text), LISTA (Library, Information Science & Technology Abstracts) via EBSCO, and the Web of Science Core Collection using database-specific search strategies. Additionally, we will perform free web searching via Google Scholar as well as direct forward and backward citation tracking of included studies. For citation tracking, we will use Scopus, as this database seems to cover the largest number of relevant citations for the purpose of our review ^30^. We will also contact librarians in the field of health sciences and information specialists through several mailing lists (e.g. Netzwerk Fachbibliotheken Gesundheit, Canadian Medical Libraries, Expertsearching, MEDBIB-L/German-speaking medical librarians, and EAHIL-list) to ask for further studies.

***Search strategy.*** Due to a lack of adequate index terms, our search strategy will be based on textwords only. To determine frequently occurring terms for inclusion into the search strategy, we analysed keywords in the titles and abstracts of potentially relevant publications retrieved from preliminary searches and similar articles identified via PubMed by using a text mining approach ^31^. We restricted parts of our textwords to the title field in order to avoid retrieving systematic reviews using citation tracking.

All authors contributed to the development of search strategies. HE and CAH are information specialists with a professional background in research; JH and TN are researchers experienced in the development of search strategies. HE drafted the search strategy. To uncover potential flaws, JH peer-checked the drafted search strategy and discussed it with HE.

Box 1 shows the final search strategy for MEDLINE in Ovid syntax. To use the strategy in other databases, we will translate it by means of Polyglot Search Translator ^32^. CAH will conduct the searches and eliminate duplicates using the Bramer method ^33^. We will perform free web searching in Google Scholar using search terms from our database search.

Box 1. **Search strategy for MEDLINE via Ovid**

(reference list OR reference lists OR ((reference OR references OR citation OR citations OR co-citation OR co-citations) ADJ3 (search OR searches OR searching OR searched OR screen OR screening OR chain OR chains OR chaining OR check OR checking OR checked OR chased OR chasing OR tracking OR tracked OR harvesting OR tool OR tools OR backward OR forward)) OR ((cited OR citing OR cocited OR cociting OR co-cited OR co-citing) ADJ3 (references OR reference)) OR citation discovery tool OR cocitation OR co-citation OR cocitations OR co-citations OR co-cited OR backward chaining OR forward chaining OR snowball sampling OR snowballing OR footnote chasing OR berry picking OR cross references OR cross referencing OR cross-references OR cross-referencing OR citation activity OR citation activities OR citation analysis OR citation analyses OR citation network OR citation networks OR citation relationship OR citation relationships).ti OR (((((strategy OR strategies OR method* OR literature OR evidence OR additional OR complementary OR supplementary) ADJ3 (find OR finding OR search* OR retriev*)) OR (database ADJ2 combin*)).ti) AND ((search OR searches OR searching OR searched).ab))

***Data management.*** A bibliography management tool (e.g. Citavi, EndNote) will be used to manage the number of reference retrievals throughout the study selection process. Furthermore, we will use specific tools for study selection that we describe below.

***Selection of sources of evidence.*** After an initial calibration phase (TN, JH, HE), two authors (JH, TN) will independently screen titles, abstracts, and full texts using Rayyan ^34^. They will solve disagreements by third author arbitration (HE). To screen the results of the citation tracking step, we will consider ASReview, particularly if the number of references exceeds 1000. ASReview combines machine (deep) learning models on a set of eligible studies with active learning on manual selections during title-abstract screening to generate a relevancy-ranked abstract list and to save screening time. Should the tool prove to be beneficial for reducing the screening load, we will consider conducting a more sensitive database search at a later stage and screen additional results with ASReview.

***Data charting process.*** We will pilot a standardised data extraction sheet approved by consensus among the authors. We will extract bibliographic and geographic data, design- and study-specific data as well as results. We aim for an iterative data extraction process, but in the final publication, we will provide a detailed overview of extracted data items. The first author (JH) will extract data and the second author (TN) will peer-check the extraction. We will solve disagreements by third author arbitration (HE).

***Synthesis of results.*** One author (JH) will narratively summarise study characteristics and results based on the data extraction. Depending on the results, we will also chart them graphically.

### Expert survey

***Setting.*** The results of the scoping review will inform our online expert survey (see below). We intend to collect data from international experts in the field of literature retrieval methods. To design and develop the survey, we will use SosciSurvey ^35^. The survey language will be English.

***Recruitment.*** Recruitment will be based on a triple approach. First, we will contact authors of pertinent articles identified during the literature search as well as experts from our professional networks. This "person-based" approach will help us to identify experts who authored papers, books, comments, and reviews in the field of citation tracking. We will ask the contacted persons to take part in the survey and to forward the invitation ^36^. Second, we will identify and contact relevant national and international networks as well as systematic review collaborations using institutional contacts (e.g. Cochrane, Joanna Briggs Institute, Campbell Collaboration, Institute of Medicine, expert information specialists, and Evidence Synthesis International). We will also use mailing lists (e.g. Netzwerk Fachbibliotheken Gesundheit, Canadian Medical Libraries, Expertsearching, MEDBIB-L/German-speaking medical librarians, and EAHIL-list). This "network-based approach" will allow us to reach experts in the field of literature retrieval methods who are potentially using citation tracking without necessarily being the authors of methodological studies (yet). Third, we will announce the expert survey on our project page on ResearchGate, aiming to recruit further experts missed by our person- and network-based approaches. By using this triple approach, we intend to recruit between 60 and 150 participants. The authors of this study will not participate in the survey.

***Data collection.*** We will iteratively develop the survey questions during the scoping review process based on our findings. Broadly speaking, our survey will contain questions on the use of citation tracking, preferred taxonomy, experiences with citation tracking, as well as recommendations for future practice and research. We will collect participantsʼ sociodemographic data, e.g. professional education and background, current field of work, years of experience in literature searching and citation tracking. We will schedule approximately five weeks for participation. Table 2 illustrates our reminder strategy to reach as many experts as possible.

**Table 2.** Reminder strategy of the online survey.

Process and time	Person-based approach	Network-based approach	Announcement on ResearchGate
Survey setup	Invitation	Invitation	Invitation
Two weeks after	Reminder	-	-
Four weeks after	Reminder	Reminder	Update
Survey closing	-	-	Closing information

Note: Person-based approach: contacting national and international networks and systematic review collaborations using mailing lists.

***Data analysis.*** To analyse the survey data, we will apply descriptive statistics based on frequencies, percentages, and cross tables. We will thematically categorise free text answers ^37^.

***Ethical concerns.*** The online expert survey will contain introductory information on our aims, the survey itself, data management and security. Since we do not expect vulnerability on the part of participants, we will not apply for ethical approval of the expert survey. Taking part in the survey will indicate consent to participate.

## Dissemination of results

Our dissemination strategy uses multiple ways to share our study results with academic stakeholders. The final review and expert survey will be published in an international open access journal relevant in the field of information retrieval. Additionally, we will discuss our results with experts at national and international conferences (e.g. conference of the German Network for Evidence-based Medicine (EbM-Netzwerk), conference of the European Association for Health Information and Libraries (EAHIL). To inform about our study results and publications, we will use Twitter, ResearchGate, and mailing lists from relevant stakeholders such as Netzwerk Fachbibliotheken Gesundheit, Canadian Medical Libraries, Expertsearching, MEDBIB-L/German-speaking medical librarians, and EAHIL-list.

## Study status

We intend to start our search in November 2020 and expect to complete the study in April 2022.

*Current study status:* preliminary searches: yes; piloting of the study selection process: yes; formal screening of search results against eligibility criteria: no; data extraction: no; data analysis: no.

## Conclusions

Depending on the available study landscape, missing pertinent evidence might have an impact on the validity of systematic reviews and, consequently, on the quality of clinical care ^38– 41^. Therefore, authors of systematic reviews should conduct high quality literature searches aiming to detect all relevant evidence. Citation tracking may be a good way to complement electronic database searches and to broaden the scope of possible findings. Therefore, our study intends to provide literature- and expert-based recommendations on the use of citation tracking for systematic literature searching. Specifically, we will provide guidance on; (i) situations in which citation tracking is likely to be particularly effective, (ii) situations in which a particular method of citation tracking is specifically indicated, and on (iii) technical/methodological aspects and pitfalls. We will also point out (iv) areas requiring more research in order to provide recommendations. It is possible that the recommendations developed during this project in a health-related context may prove relevant also for other academic fields such as social or environmental sciences ^9, 42^. Finally, tailored and evidence-based recommendations concerning the use of citation tracking for systematic literature searching may guide future steps in semi-automated and automated literature retrieval methods ^43, 44^.

## Data availability

### Underlying data

No underlying data are associated with this article.

### Reporting guidelines

Open Science Framework (OSF): PRISMA-P checklist for ‘Using citation tracking for systematic literature searching: study protocol for a scoping review of methodological studies and an expert survey’, https://doi.org/10.17605/OSF.IO/7ETYD ^23^.

Data are available under the terms of the Creative Commons Zero "No rights reserved" data waiver (CC0 1.0 Public domain dedication).
