# Using citation tracking for systematic literature searching - study protocol for a scoping review of methodological studies and a Delphi study

**DOI:** 10.12688/f1000research.27337.2

**Published:** 2021-08-04

**Authors:** Julian Hirt, Thomas Nordhausen, Christian Appenzeller-Herzog, Hannah Ewald

**Affiliations:** 1 Institute of Applied Nursing Science, Department of Health, Eastern Switzerland University of Applied Sciences (formerly FHS St.Gallen), St.Gallen, Switzerland; 2 International Graduate Academy, Institute of Health and Nursing Science, Medical Faculty, Martin Luther University Halle-Wittenberg, Halle (Saale), Germany; 3 Department of Clinical Research, University Hospital Basel, University of Basel, Basel, Switzerland; 4 University Medical Library, University of Basel, Basel, Switzerland

**Keywords:** Citation Tracking, Literature Search, Supplementary Search, Methods, Scoping Review, Research Methodology, Survey, Systematic Review

## Abstract

**Background:** Up-to-date guidance on comprehensive study identification for systematic reviews is crucial. According to current recommendations, systematic searching should combine electronic database searching with supplementary search methods. One such supplementary search method is citation tracking. It aims at collecting directly and/or indirectly cited and citing references from "seed references". Tailored and evidence-guided recommendations concerning the use of citation tracking are strongly needed.

**Objective:** We intend to develop recommendations for the use of citation tracking in systematic literature searching for health-related topics. Our study will be guided by the following research questions: What is the benefit of citation tracking for systematic literature searching for health-related topics? Which methods, citation indexes, and other tools are used for citation tracking? What terminology is used for citation tracking methods?

**Methods:** Our study will have two parts: a scoping review and a Delphi study. The scoping review aims at identifying methodological studies on the benefit and use of citation tracking in systematic literature searching for health-related topics with no restrictions on study design, language, and publication date. We will perform database searching in MEDLINE (Ovid), CINAHL (EBSCOhost), Web of Science Core Collection, two information science databases, web searching, and contact experts in the field. Two reviewers will independently perform study selection. We will conduct direct backward and forward citation tracking on included articles. Data from included studies will be extracted using a prespecified extraction sheet and presented in both tabular and narrative form. The results of the scoping review will inform the subsequent Delphi study through which we aim to derive consensus recommendations for the future practice and research of citation tracking.

## Introduction

Systematic reviews are considered to be of high clinical and methodological importance as they help to derive recommendations for health care practice and future research ^1– 3^. A comprehensive literature search that aims to identify the available evidence as completely as possible is the foundation of every systematic review ^4– 6^. Due to an ever-growing research volume, lack of universal terminology and indexation, as well as extensive time requirements for identifying studies in a systematic way, efficient search approaches are required ^5, 7, 8^. According to current recommendations, systematic search approaches should include both electronic database searching and one or several supplementary search methods ^9^. Potential supplementary search methods include citation tracking, contacting study authors or experts, handsearching, trial register searching, and web searching ^10^. In this study, we focus on citation tracking.

**Figure 1.**
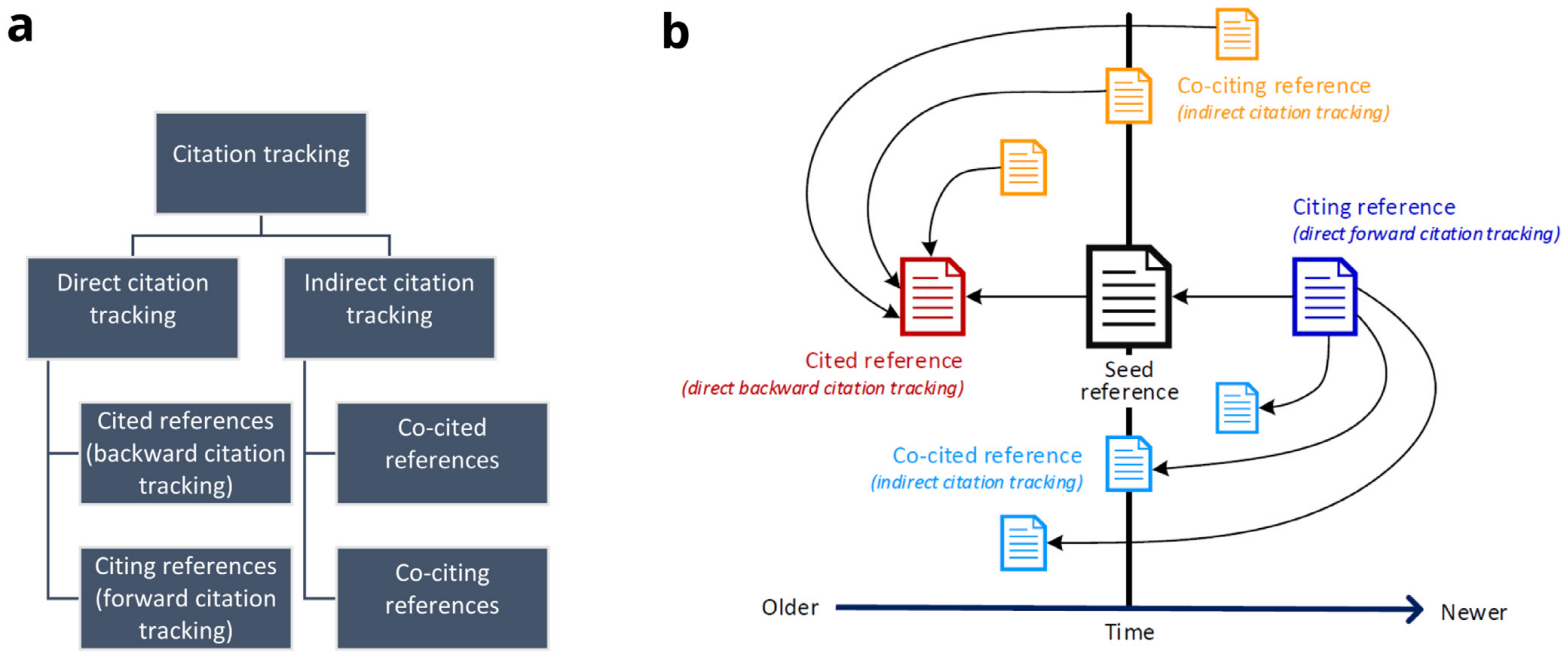
Overview of citation tracking methods. **1a**: Hierarchical illustration of different citation tracking methods; **1b**: Direct and indirect citation relationships of references based on a seed reference. A → B denotes A cites B. The horizontal axis denotes time, i.e. the chronology in which references were published relative to the seed reference: “Older” stands for references that were published before the seed reference, “Newer” stands for references that were published after the seed reference.

Citation tracking is an umbrella term for multiple methods which directly or indirectly collect related references from so called "seed references". These seed references are usually eligible for inclusion into the review. Some may be known at the beginning of the review and others may emerge as eligible records following full-text screening ^10– 12^. The terminology used to describe the principles of citation tracking is non-uniform and heterogeneous ^13– 16^. Citation tracking methods are sub-categorized into *direct* and *indirect* citation tracking ( Figure 1a). For direct citation tracking, the words "backward" and "forward" denote the directionality of tracking ^13, 17, 18^. Backward citation tracking is the oldest form of citation tracking. It aims at identifying references cited by a seed reference - which can easily be achieved by checking the reference list. Terms like "footnote chasing" or "reference list searching" are synonyms ^6, 13^. In contrast, forward citation tracking or chaining aims at identifying citing references, i.e. references that cite a seed reference ^19^. *Indirect* citation tracking describes the identification of (i) co-cited references or co-citations (i.e. other references cited by citing literature of a seed reference) and of (ii) co-citing references (i.e. publications sharing references with a seed reference) ^11, 20^. Direct and indirect citation relationships of references based on a seed reference are illustrated in Figure 1b. Both direct and indirect citation tracking may contain one or more layers of iteration. To this end, researchers may use newly retrieved, relevant references as new seed references.

Direct backward citation tracking of cited references is currently the most common citation tracking method. However, recent guidance suggests that a combination of several methods (e.g. tracking cited, citing, co-cited and co-citing references) may be the most effective way to use citation tracking for systematic reviewing ^10^. It is quite likely that the added value of any form of citation tracking is not the same for all systematic reviews. It rather depends on a variety of factors. For instance, citation tracking may be beneficial in research areas that require complex searches such as reviews of complex interventions, mixed-methods reviews, qualitative evidence syntheses, or reviews on public health topics. Furthermore, research areas without consistent terminology or with vocabulary overlaps with other fields, such as methodological topics, may also benefit from the use of citation tracking ^20, 21^. Hence, tailored and evidence-guided recommendations on the use of citation tracking are strongly needed. However, none of the current reviews on this topic has systematically identified available evidence on the use and benefit of citation tracking in the context of systematic literature searching ^10^.

Therefore, the aim of our study is to develop recommendations for the use of citation tracking in systematic literature searching for health-related topics. The scoping review will be guided by the following three research questions which in turn will inform the Delphi study:

- What is the benefit of citation tracking for systematic literature searching for health-related topics?
- Which methods, citation indexes, and other tools are used for citation tracking?
- What terminology is used for citation tracking methods?

## Protocol

This protocol is reported according to the “Preferred Reporting Items for Systematic review and Meta-Analysis Protocols” (PRISMA-P) checklist ^22^ which we published on the Open Science Framework ^23^. Our study will have two parts: a scoping review and a Delphi study. The scoping review has the objective to map the benefit and the use of citation tracking, or research gaps if the results are not sufficiently informative. The objective of the subsequent Delphi study is to derive consensus recommendations for future practice and research of citation tracking ^24– 26^. For the scoping review, we will use the framework by Arksey and O’Malley ^26^ and the “Preferred Reporting Items for Systematic reviews and Meta-Analyses extension for Scoping Reviews” (PRISMA-ScR) ^27^. For the Delphi study, we will follow the “Guidance on Conducting and REporting DElphi Studies” (CREDES) statement ^28^.

### Scoping review

***Eligibility criteria.*** We will include any study with a focus on citation tracking as a means of evidence retrieval which exhibits one of the following criteria: benefit and/or effectiveness of (i) citation tracking in general; (ii) different methods of citation tracking (e.g. backward vs. forward, direct vs. indirect); or (iii) technical uses of citation tracking (e.g. comparing citation indexes and/or tools, e.g. Scopus vs. Web of Science, Oyster, Voyster). Eligible studies need to have a health-related context. There will be no restrictions on study design, language, and publication date.

We will exclude studies solely using citation tracking for evidence retrieval, e.g. a systematic review applying citation tracking as a supplementary search technique, or studies focussing on citation tracking as a means to explore network or citation impact (i.e. bibliometric analysis). Studies only assessing the benefit of combined search methods in which the isolated benefit of citation tracking cannot be extracted will also be excluded. Furthermore, we will exclude methodological guidelines without empirical investigations and other non-empirical publications like editorials, commentaries, letters and abstract-only publications. Table 1 illustrates our inclusion and exclusion criteria per domain.

**Table 1.** Inclusion and exclusion criteria.

Domain	Inclusion criteria	Exclusion criteria
Study focus	Any study with a focus on citation tracking as an evidence retrieval method AND one of the following criteria: - any study assessing the benefit and/or effectiveness of citation tracking - any study comparing different methods of citation tracking (e.g. backward vs. forward, direct vs. indirect) - any study assessing technical uses of citation tracking (e.g. comparing citation indexes and/or tools, e.g. Scopus vs. WoS, Oyster, Voyster, etc.)	Any study solely using citation tracking for evidence retrieval (e.g. a systematic review applying citation tracking as supplementary search technique) OR any study solely assessing benefits and/or use and/or effectiveness of citation tracking to explore a network or citation impact (i.e. bibliometric analysis) OR any study describing solely the method of citation tracking without further assessing it, e.g. guidelines for developing search strategies or guidelines for systematic or other reviews OR any study only assessing the benefit of combined search methods in which the isolated benefit of citation tracking cannot be extracted
Research context	Health-related	Other
Language	All languages	-
Publication year	All publication years	-
Publication type	Any reports of empirical studies	Editorials Commentaries Letters Abstract-only publications

***Information sources.*** We will search MEDLINE via Ovid; CINAHL (Cumulative Index to Nursing and Allied Health Literature), LLISFT (Library Literature & Information Science Full Text) and LISTA (Library, Information Science & Technology Abstracts) via EBSCOhost, and the Web of Science Core Collection by using database-specific search strategies. Additionally, we will perform web searching via Google Scholar as well as direct forward and backward citation tracking of included studies. As some evidence suggests that one citation index may not be enough for this ^29^, we will use Scopus, Web of Science, and Google Scholar for forward citation tracking. For backward citation tracking, we will use Scopus and, if seed references are not indexed in Scopus, we will manually extract the seed reference's reference list. We will iteratively repeat direct citation tracking on newly identified eligible references until no further eligible references will be identified. We will also contact librarians in the field of health sciences and information specialists through several mailing lists (Canadian Medical Libraries, Expertsearching, MEDIBIB-L/German-speaking medical librarians, and EAHIL-list) to ask for further studies.

***Search strategy.*** Due to a lack of adequate index terms, our search strategy will be based on text words only. To determine frequently occurring terms for inclusion into the search strategy, we analysed keywords in the titles and abstracts of potentially relevant publications retrieved from preliminary searches and similar articles identified via PubMed by using various text mining tools ( PubMed Reminer, AntConc, Yale MeSH analyzer, Voyant, VOSviewer, Termine, Text analyzer) ^30^. We restricted some of our text words to the title field in order to avoid retrieving systematic reviews that used citation tracking.

All authors contributed to the development of search strategies. HE and CAH are information specialists with a professional background in research; JH and TN are researchers experienced in the development of search strategies. HE drafted the search strategy and JH peer-checked it.

Box 1 shows the final search for MEDLINE in Ovid syntax. To use the search in other databases, we will translate it by means of Polyglot Search Translator ^31^. CAH will conduct the searches and eliminate duplicates using the Bramer method ^32^. We will perform web searching in Google Scholar using search terms from our database search. We will document our search strategy according to PRISMA-S ^33^.

Box 1. **Search strategy for MEDLINE via Ovid**

(reference list OR reference lists OR ((reference OR references OR citation OR citations OR co-citation OR co-citations) ADJ3 (search OR searches OR searching OR searched OR screen OR screening OR chain OR chains OR chaining OR check OR checking OR checked OR chased OR chasing OR tracking OR tracked OR harvesting OR tool OR tools OR backward OR forward)) OR ((cited OR citing OR cocited OR cociting OR co-cited OR co-citing) ADJ3 (references OR reference)) OR citation discovery tool OR cocitation OR co-citation OR cocitations OR co-citations OR co-cited OR backward chaining OR forward chaining OR snowball sampling OR snowballing OR footnote chasing OR berry picking OR cross references OR cross referencing OR cross-references OR cross-referencing OR citation activity OR citation activities OR citation analysis OR citation analyses OR citation network OR citation networks OR citation relationship OR citation relationships).ti OR (((((strategy OR strategies OR method* OR literature OR evidence OR additional OR complementary OR supplementary) ADJ3 (find OR finding OR search* OR retriev*)) OR (database ADJ2 combin*)).ti) AND ((search OR searches OR searching OR searched).ab))

***Data management.*** A bibliography management tool will be used to manage the number of reference retrievals throughout the study selection process. Furthermore, we will use specific tools for study selection that we describe below.

***Selection of sources of evidence.*** After an initial calibration phase, that is screening 100 titles and abstracts separately and discussing divergent decisions (TN, JH, HE), two authors (JH, TN) will independently screen titles, abstracts, and full texts using Rayyan ^34^. They will solve disagreements by third author arbitration (HE). To screen the results of the citation tracking step, we will consider ASReview, particularly if the number of references exceeds 1000. ASReview combines machine (deep) learning models on a set of eligible studies with active learning on manual selections during title-abstract screening to generate a relevancy-ranked abstract list and to save screening time. Should the tool prove to be beneficial for reducing the screening load, we will consider conducting a more sensitive database search at a later stage and screen additional results with ASReview.

***Data charting process.*** We will pilot a prespecified data extraction sheet approved by consensus among the authors. We will extract bibliographic and geographic data, design- and study-specific data as well as results that answer our research questions. Since we expect heterogeneous studies in terms of aim, design, and methods, we aim for an iterative data extraction process. This will allow a flexible and study-specific data extraction process, e.g. by adding previously neglected data extraction items that might contribute to the overall body of knowledge to the data extraction form. In the final publication, we will provide a detailed overview of extracted data items. One author will extract data and a second author will peer-check the extraction. We will solve disagreements by third author arbitration.

***Synthesis of results.*** One author (JH) will narratively summarise study characteristics and results. Depending on the results, we will also chart them graphically.

### Delphi study

***Design and rationale.*** A consensus multi-stage online Delphi procedure will be used to derive recommendations for the use of citation tracking in systematic literature searching for health-related topics ^29^. A Delphi procedure will be chosen since the method enables to collect the perspectives of international experts on citation tracking, promote discussions on the topic as well as derive consensus recommendations for future practice and research. The Delphi study will entail several Delphi rounds. The results of the scoping review will inform the initial Delphi round (see below for details). To distribute the Delphi rounds to the experts, we will use the web-based tool SosciSurvey ^35^. The Delphi language will be English.

***Expert panel.*** The recruitment of experts will be based on a stepwise approach. First, we will contact authors of pertinent articles identified during the literature search as well as experts from our professional networks. This "person-based" approach will help us to identify experts who authored papers, books, comments, and reviews in the field of citation tracking. We will ask the contacted persons to take part in the Delphi study ^36^. Second, we will identify and contact relevant national and international organisations as well as systematic review collaborations (e.g. Cochrane groups, Joanna Briggs Institute (JBI), Campbell Collaboration, National Academy of Medicine (NAM), expert information specialists, Evidence Synthesis International, and PRISMA-S working group). This "organisation-based approach" will allow us to reach experts in the field of literature retrieval methods who are potentially using citation tracking without necessarily being the authors of methodological studies (yet). By using this stepwise approach, we intend to recruit at least 15 experts.

***Data collection.*** Throughout our online Delphi rounds, we will seek guidance on various aspects of citation tracking. For example, recommendations on the following aspects could be of particular interest:

- Uniform terminology for citation tracking methods
- Situations in which citation tracking should be applied
- Potential situations in which citation tracking can be used as a sole method of evidence retrieval
- Situations in which a particular citation tracking method or a combination thereof is likely to be most effective
- Situations in which further layers of iteration of citation tracking should be applied
- Necessity to use multiple citation indexes for citation tracking
- For indirect citation tracking, screening of selected records only and definition of their ranking and cut-off
- Reporting of citation tracking (complementing PRISMA-S ^33^)
- Questions on citation tracking that currently cannot be answered and require more research

Based on the results of our scoping review, we will formulate draft recommendations for the first round of Delphi. However, if according to the scoping review, pertinent evidence is missing, we will ask for the experiences and perspectives of the Delphi experts to initiate the consensus process.

To describe experts’ characteristics, we will collect sociodemographic data, i.e. professional education and background, current field of work as well as years of experience in literature searching and citation tracking. We assume that several Delphi rounds will be needed to derive consensus recommendations for the use of citation tracking. We expect that experts will invest around 30 to 90 minutes per Delphi round depending on the underpinning aim of the Delphi round as well as experts’ familiarity and experiences with the topic. For each Delphi round, we will schedule approximately three weeks for participation. Table 2 illustrates our reminder strategy within a Delphi round. We will pilot test and discuss our Delphi items with a person experienced in literature searching who is not an author and not involved in the Delphi study.

**Table 2.** Reminder strategy of each online Delphi round.

Process and time	Person-based approach	Organisation-based approach
Delphi round setup	Invitation	Invitation
One week after	Reminder	-
Two weeks after	Reminder	Reminder
Delphi round closing after three weeks	-	-

Note: Person-based approach: contacting authors of pertinent articles identified during the literature search as well as experts from authors’ professional networks; Organisation-based approach: contacting national and international organisations and systematic review collaborations.

***Data analysis.*** For free text answers and statements of experts, we will use thematic categorisation ^37^. For votes whose results are available or can be converted into numbers, we will use descriptive statistics.

***Ethical concerns.*** The online Delphi study will contain introductory information on our aims, the Delphi itself, data management and security. We do not expect vulnerability on the part of experts and with regard to the Swiss Human Research Act, our research does not concern human diseases and the structure and function of the human body ^38^. We will therefore not apply for ethical approval of the Delphi study. Taking part in the Delphi study will indicate consent to participate. There will be no mandatory participation once an expert consented to participate. Experts will not receive an incentive for participation and may leave the process at any time.

## Dissemination of results

Our dissemination strategy uses multiple ways to share our study results with academic stakeholders. The final scoping review and Delphi study will each be published in an international open access journal relevant in the field of information retrieval. Additionally, we will discuss our results with experts at national and international conferences (e.g. conference of the German Network for Evidence-based Medicine (EbM-Netzwerk), conference of the European Association for Health Information and Libraries (EAHIL), Cochrane Colloquium, Health Technology Assessment International (HTAi) conference). To inform about our study results and publications, we will use Twitter, ResearchGate, and mailing lists from relevant stakeholders such as Canadian Medical Libraries, Expertsearching, MEDIBIB-L/German-speaking medical librarians, and EAHIL-list.

## Study status

We intend to start our search in November 2020 and expect to complete the study in 2022.

*Current study status:* preliminary searches: yes; piloting of the study selection process: yes; formal screening of search results against eligibility criteria: yes; data extraction: no; data analysis: no.

## Conclusions

Missing pertinent evidence might have an impact on the validity of systematic reviews and, consequently, on the quality of health care ^39, 40^. Therefore, authors of systematic reviews should conduct high quality literature searches aiming to detect all relevant evidence. Citation tracking may be an effective way to complement electronic database searches and to broaden the scope of possible findings. Therefore, our study intends to provide literature- and expert-based recommendations on the use of citation tracking for systematic literature searching. Although we solely focus on a health-related context, it is possible that some of the recommendations developed during this project may prove relevant also for other academic fields such as social or environmental sciences ^9, 41^. Finally, tailored and evidence-based recommendations concerning the use of citation tracking for systematic literature searching may guide future steps in semi-automated and automated literature retrieval methods ^42, 43^.

## Data availability

### Underlying data

No underlying data are associated with this article.

### Reporting guidelines

Open Science Framework (OSF): PRISMA-P checklist for ‘Using citation tracking for systematic literature searching - study protocol for a scoping review of methodological studies and a Delphi study’, https://doi.org/10.17605/OSF.IO/7ETYD ^23^.

Data are available under the terms of the Creative Commons Zero "No rights reserved" data waiver (CC0 1.0 Public domain dedication).
