# Supplementary material for: Using citation tracking for systematic literature searching - study protocol for a scoping review of methodological studies and a Delphi study
Source: F1000Res. 2021 Sep 2;9:1386. Originally published 2020 Dec 1. [Version 3] doi: 10.12688/f1000research.27337.3 (PMC8474097; doi:10.12688/f1000research.27337.3)
Supplement: Peer review history

# Peer review: Reviewer response for version 3

**DOI:** 10.5256/f1000research.77208.r93320

**Published:** 2021-09-23

**Authors:** David Moher

**Affiliations:** 1 School of Epidemiology and Public Health, University of Ottawa, Ottawa, Canada; 2 Centre for Journalology, Ottawa Hospital Research Institute, Ottawa, Canada

The authors have addressed the concerns I raised in my second review.

Is the study design appropriate for the research question?

Yes

Is the rationale for, and objectives of, the study clearly described?

Yes

Are sufficient details of the methods provided to allow replication by others?

Partly

Are the datasets clearly presented in a useable and accessible format?

Partly

Reviewer Expertise:

Systematic reviews; open science; reporting guidelines.

I confirm that I have read this submission and believe that I have an appropriate level of expertise to confirm that it is of an acceptable scientific standard.

# Peer review: Reviewer response for version 2

**DOI:** 10.5256/f1000research.55984.r91228

**Published:** 2021-08-18

**Authors:** Julie Glanville

**Affiliations:** 1 Cochrane Information Retrieval Methods Group, London, UK

I have read the revised version of the study protocol and I am happy with the changes that have been made.

Is the study design appropriate for the research question?

Yes

Is the rationale for, and objectives of, the study clearly described?

Yes

Are sufficient details of the methods provided to allow replication by others?

Partly

Are the datasets clearly presented in a useable and accessible format?

Not applicable

Reviewer Expertise:

Information retrieval for evidence identification for systematic reviews.

I confirm that I have read this submission and believe that I have an appropriate level of expertise to confirm that it is of an acceptable scientific standard.

# Peer review: Reviewer response for version 2

**DOI:** 10.5256/f1000research.55984.r91227

**Published:** 2021-08-18

**Authors:** David Moher

**Affiliations:** 1 School of Epidemiology and Public Health, University of Ottawa, Ottawa, Canada; 2 Centre for Journalology, Ottawa Hospital Research Institute, Ottawa, Canada

I think the revisions, particularly around the scoping review, are much improved.

 In terms of the Delphi study, I think details are still missing. For example, it is unclear how many rounds will be given. After round 1, what consensus rate (e.g., 75%) will be used to drop an item from subsequent rounds? Overall, I think more details are required for the proposed Delphi methods. My recommendation is to read these papers:

- Examples of Delphi studies: Vogel *et al*., 2019 ^1^, Santaguida *et al*., 2018 ^2^;
- General methodological issues about Delphi: Okoli *et al*., 2004 ^3^, Diamond *et al*., 2014 ^4^

One final minor detail. In the study status section of the paper, the authors state they started in November 2020. Is this correct?

Is the study design appropriate for the research question?

Yes

Is the rationale for, and objectives of, the study clearly described?

Yes

Are sufficient details of the methods provided to allow replication by others?

Partly

Are the datasets clearly presented in a useable and accessible format?

Partly

Reviewer Expertise:

Systematic reviews; open science; reporting guidelines.

I confirm that I have read this submission and believe that I have an appropriate level of expertise to confirm that it is of an acceptable scientific standard, however I have significant reservations, as outlined above.

## References

[1] : A Delphi study to build consensus on the definition and use of big data in obesity research.*Int J Obes (Lond)*.43(12) : 10.1038/s41366-018-0313-92573-258610.1038/s41366-018-0313-930655580PMC6892733

[2] : Protocol for a Delphi consensus exercise to identify a core set of criteria for selecting health related outcome measures (HROM) to be used in primary health care. *BMC Family Practice*.2018;19(1) : 10.1186/s12875-018-0831-510.1186/s12875-018-0831-530185172PMC6123958

[3] : The Delphi method as a research tool: an example, design considerations and applications. *Information & Management*.2004;42(1) : 10.1016/j.im.2003.11.00215-2910.1016/j.im.2003.11.002

[4] : Defining consensus: a systematic review recommends methodologic criteria for reporting of Delphi studies.*J Clin Epidemiol*.2014;67(4) : 10.1016/j.jclinepi.2013.12.002401-910.1016/j.jclinepi.2013.12.00224581294

# Response

**Published:** 2021-08-29

**Authors:** Julian Hirt

**Affiliations:** University Hospital Basel, Switzerland

*Dear David Moher*

*Thank you very much for your helpful comments and references. We revised our manuscript with respect to methodological details of the Delphi study and now specified the number of rounds we expect to perform, the consensus rate, anonymity of participants, and expected non-response.*

 Reviewer comment: I think the revisions, particularly around the scoping review, are much improved.

 In terms of the Delphi study, I think details are still missing. For example, it is unclear how many rounds will be given. After round 1, what consensus rate (e.g., 75%) will be used to drop an item from subsequent rounds? Overall, I think more details are required for the proposed Delphi methods. My recommendation is to read these papers:

 Examples of Delphi studies: Vogel et al., 2019 1, Santaguida et al., 2018 2;

 General methodological issues about Delphi: Okoli et al., 2004 3, Diamond et al., 2014 4

*Authors’ response: We added “Based on the results of our scoping review, we will formulate draft recommendations for the first Delphi round. Experts will be invited to rate their agreement with the draft recommendations using a four-point Likert scale (strongly agree – agree – disagree – strongly disagree). If experts vote disagree/strongly disagree, they will be required to comment on their reasons and/or give constructive feedback. We consider a recommendation as consented when at least 75% of the experts agree/strongly agree. All other recommendations will be adapted for the next Delphi round. This adaptation will be based on the comments collected from the experts and, if necessary, on discussion via video conference.*

*There are items where we will not directly propose recommendations, e.g., if the results of our scoping review do not allow it or if there are several equally valid options (e.g., for terminology). In these cases, we will either ask the Delphi experts for their experiences and perspectives or let them vote on several options. We will use the resulting answers to formulate draft recommendations, which will be entered into the Delphi consensus process (see above). Therefore, our Delphi study may comprise qualitative and quantitative aspects.*

*We will limit the number of Delphi rounds to a maximum of four rounds. Should there be no consensus for any of the items by the end of the fourth round, we will report the results but not give any recommendations.*

*Expert assessments will be anonymous among experts but open to the study team. We expect a low non-response rate since experts' participation is indicative of their interest in our study.”*

 One final minor detail. In the study status section of the paper, the authors state they started in November 2020. Is this correct?

*Authors’ response: Yes, we started to work on this scoping review in November by doing the initial searches. In the revised version, we reworded to be clearer on that issue: “We conducted the initial search for the scoping review in November 2020 and expect to complete the Delphi study in 2022.”*

# Peer review: Reviewer response for version 1

**DOI:** 10.5256/f1000research.30208.r81307

**Published:** 2021-04-07

**Authors:** David Moher

**Affiliations:** 1 School of Epidemiology and Public Health, University of Ottawa, Ottawa, Canada; 2 Centre for Journalology, Ottawa Hospital Research Institute, Ottawa, Canada

The authors are proposing a new guidance on citation tracking. They will achieve this by completing a scoping review and subsequently a survey. The authors propose using appropriate methods (and reporting) for conducting the scoping review. I found the methods for the survey less clear and hope my comments/questions, below, are helpful in revised the protocol.

**Questions/comments:**

1. A more conceptual question is to what degree this initiative overlaps and differs from PRISMA-S (Rethlefsen ML, Kirtley S, Waffenschmidt S, Ayala AP, Moher D, Page MJ, Koffel JB; PRISMA-S Group. PRISMA-S: an extension to the PRISMA Statement for Reporting Literature Searches in Systematic Reviews. Syst Rev. 2021 Jan 26;10(1):39. doi: 10.1186/s13643-020-01542-z) ^1^.
2. For example, item 5 of PRIMSA-S – Citation searching - states “Citation searching 5 Indicate whether cited references or citing references were examined, and describe any methods used for locating cited/citing references (e.g., browsing reference lists, using a citation index, setting up email alerts for references citing included studies).” I think this is important so as not to potentially confuse readers. Indeed, should the current initiative be seen as an implementation of PRISMA-S?
3. I had more difficulty understanding the proposed survey. For me, much of the methods were missing. I understand the recruitment (well reported). I would recommend the authors include the PRISMA-S group unless there is a lot of overlap between PRISMA-S and the other groups mentioned.
4. It was not clear to me how long the authors will make completing the survey– 15 minutes of 45 minutes? Similarly, will the respondents receive an incentive for completing the survey?
5. Why ‘simply’ a survey rather than a Delphi (or modified) approach? Will the survey (or Delphi) be pilot tested for question and response option clarity and language?
6. The data analysis describes cross tables. I assume the authors mean cross tabulations which can be more than descriptive. Will the authors be conducting Chi-square analysis or other analytical approaches of the cross tabulations (e.g., p values)?
7. The ethical concerns section is likely jurisdiction specific. In my setting ethics would be required. Can the authors explicitly indicate whether ethics is required or not. The section is currently vague on this critical issue. 
8. For the survey, do the authors have an estimated sample size they are aiming for. Similarly, do the authors have an estimated response rate they are aiming for?

Is the study design appropriate for the research question?

Yes

Is the rationale for, and objectives of, the study clearly described?

Yes

Are sufficient details of the methods provided to allow replication by others?

Partly

Are the datasets clearly presented in a useable and accessible format?

Partly

Reviewer Expertise:

Systematic reviews; open science; reporting guidelines.

I confirm that I have read this submission and believe that I have an appropriate level of expertise to confirm that it is of an acceptable scientific standard, however I have significant reservations, as outlined above.

## References

[1] : PRISMA-S: an extension to the PRISMA Statement for Reporting Literature Searches in Systematic Reviews.*Syst Rev*.2021;10(1) : 10.1186/s13643-020-01542-z3910.1186/s13643-020-01542-z33499930PMC7839230

# Response

**Published:** 2021-07-09

**Authors:** Julian Hirt

**Affiliations:** University Hospital Basel, Switzerland

Dear David Moher

 We are grateful for your reviewer report and, in particular, for your helpful suggestions to derive evidence-based recommendations from our study. Below, we provide a detailed point-by-point response with changes that we implemented in version 2 of our manuscript.

 You will see that in response to your comments, we replaced the planned survey with a planned Delphi study to address the original objective. This change also entails the reformulation of our research questions and the intent to publish the final scoping review and the results of the Delphi study separately.

 We hope that these changes fully meet your concerns.

 Sincerely yours,

 Julian Hirt, Thomas Nordhausen, Christian Appenzeller-Herzog, and Hannah Ewald

*Reviewer comment: A more conceptual question is to what degree this initiative overlaps and differs from PRISMA-S (Rethlefsen ML, Kirtley S, Waffenschmidt S, Ayala AP, Moher D, Page MJ, Koffel JB; PRISMA-S Group. PRISMA-S: an extension to the PRISMA Statement for Reporting Literature Searches in Systematic Reviews. Syst Rev. 2021 Jan 26;10(1):39. doi: 10.1186/s13643-020-01542-z).*

 Authors’ response: PRISMA-S is a reporting guideline for systematic literature searches. However, it is lacking on precise recommendations how to report citation tracking methods and results. By conducting a Delphi study with selected experts in the field (see our answer below for details), we aim at developing more detailed recommendations on adequately reporting citation tracking methods as part of systematic literature searching. This may complement recommendations on reporting given by the PRISMA-S group or could even be integrated in future versions. For the Delphi, we will invite the PRISMA-S group to best use synergies and experiences.

*Reviewer comment: For example, item 5 of PRIMSA-S – Citation searching - states “Citation searching 5 Indicate whether cited references or citing references were examined, and describe any methods used for locating cited/citing references (e.g., browsing reference lists, using a citation index, setting up email alerts for references citing included studies).” I think this is important so as not to potentially confuse readers. Indeed, should the current initiative be seen as an implementation of PRISMA-S?*

 Authors’ response: See answer above. An important detail for the reporting of our study: since we will use citation tracking as part of our information retrieval, we will use PRISMA-S to guide our reporting of the scoping review. In the revised version, we reference it accordingly: “We will document our search strategy according to PRISMA-S”.

*Reviewer comment: I had more difficulty understanding the proposed survey. For me, much of the methods were missing. I understand the recruitment (well reported). I would recommend the authors include the PRISMA-S group unless there is a lot of overlap between PRISMA-S and the other groups mentioned.*

 Authors’ response: As detailed further below, the survey will be replaced by a Delphi study. So, we replaced the survey methods with more detailed methods for the Delphi study. Concerning PRISMA-S, we agree with the reviewer and will ask the PRISMA-S working group to participate in our Delphi study (see below).

*Reviewer comment: It was not clear to me how long the authors will make completing the survey– 15 minutes of 45 minutes? Similarly, will the respondents receive an incentive for completing the survey?*

 Authors’ response: Following your recommendation below, we now plan to conduct an expert Delphi study containing multiple Delphi rounds. We expect that experts will invest around 30 to 90 minutes per Delphi round depending on the underpinning aim of the Delphi round as well as experts’ familiarity and experiences with the topic. Participants will not receive an incentive for participation.

*Reviewer comment: Why ‘simply’ a survey rather than a Delphi (or modified) approach? Will the survey (or Delphi) be pilot tested for question and response option clarity and language?*

 Authors’ response: This is an important point and we thank the reviewer for raising it. We agree that a Delphi study containing several Delphi rounds is suitable to collect the perspectives of international experts on citation tracking, promote discussions on the topic as well as to derive consensus recommendations for future practice and research on the use of citation tracking in systematic literature searching for health-related topics. We subjected our protocol to a major revision based on this point and now outline the methods for the Delphi. As stated in the revised text, we will pilot test and discuss our Delphi items with a person experienced in literature searching but who is not an author and not involved in the Delphi study.

*Reviewer comment: The data analysis describes cross tables. I assume the authors mean cross tabulations which can be more than descriptive. Will the authors be conducting Chi-square analysis or other analytical approaches of the cross tabulations (e.g., p values)?*

 Authors’ response: We revised the corresponding section to address a suitable type of analysis for our data retrieved in Delphi rounds. For free text answers and statements of experts, we will use thematic categorisation. For votes whose results are available or can be converted into numbers, we will use descriptive statistics.

*Reviewer comment: The ethical concerns section is likely jurisdiction specific. In my setting ethics would be required. Can the authors explicitly indicate whether ethics is required or not. The section is currently vague on this critical issue.*

 Authors’ response: We added details to the ethical concerns section. With regard to the Swiss Human Research Act, our research does not concern human diseases and the structure and function of the human body. We will therefore not apply for ethical approval of the expert Delphi study.

*Reviewer comment: For the survey, do the authors have an estimated sample size they are aiming for. Similarly, do the authors have an estimated response rate they are aiming for?*

 Authors’ response: This answer concerns the recruitment of experts for the Delphi study, not the survey. We intend to recruit at least 15 participants by using our stepwise approach for recruitment, the person-based approach (contacting authors of pertinent articles identified during the literature search as well as experts from authors’ professional networks) and the organisation-based approach (contacting national and international organisations and systematic review collaborations).

# Peer review: Reviewer response for version 1

**DOI:** 10.5256/f1000research.30208.r76282

**Published:** 2021-01-05

**Authors:** Julie Glanville

**Affiliations:** 1 Cochrane Information Retrieval Methods Group, London, UK

The authors propose to conduct a scoping review of the use of citation tracking techniques to identify research evidence to inform systematic reviews and also to survey experts on their use of citation tracking.

 My suggestions for action on the proposal are as follows:

1. Introduction: 'These references are usually eligible for inclusion into the review and known at the start of the citation search 10– 12.'  I think this could be reworded for clarity along the following lines: 
  'These references are usually eligible for inclusion into the review and some may be known at the beginning of the review and others may emerge as eligible records following the main database searches 10– 12.'
  Then perhaps you may not need the sentence that follows the one I am commenting on.
2. Introduction: 'The taxonomy used to describe the…'. I think it might be better to use a word such as  'terminology' rather than 'taxonomy'. 
3. Figure 1. The figure would be more helpful if it could show the relationships of the indirect citation tracking in the Figure 1b perhaps by colour coding. Please check diagrams in the following references which incorporate chronology into the  citation picture as well as showing the indirect citation tracking clearly.
  Belter CW. A relevance ranking method for citation-based search results. Scientometrics. 2017;112(2):731-46. ^1^
  Janssens A, Gwinn M, Brockman JE, Powell K, Goodman M. Novel citation-based search method for scientific literature: a validation study. BMC Med Res Methodol. 2020;20(1):25. ^2^
4. Introduction: 'However, recent guidance suggests that combining several citation tracking methods (e.g. screening cited, citing, co-cited and co-citing references) may be the most effective way to use citation tracking for systematic reviewing. '  Please cite the guidance. 
5. Introduction: ' It rather depends on a variety of factors. For instance, citation tracking may be especially beneficial in case of (i) complex searches (e.g. for reviews on public health topics), (ii) searching for health outcome measurement instruments, or (iii) research areas without consistent taxonomy, with vocabulary overlaps with other fields, or with a lack of index terms in databases (e.g. methodological topics)'.  These are all important issues and each deserves a bit more description so that readers can understand the differences. Again I think the word 'taxonomy' should be replaced with 'terminology'. 
6. Introduction: current 'topical reviews' - suggest replacing with 'recent reviews on this topic'. Then please add in more citations so that 'reviews' can be supported with more than one reference.  
7. Introduction: 'health-related systematic literature searching'.  This occurs three times and for each occurrence I suggest rewording as follows for clarity: 'literature searching for systematic reviews of health and health-related topics'. 
8. Eligibility criteria. 'evidence retrieval method'.  Suggest that 'method' is not needed as the sentence has 'means'. 
9. 'Eligible studies need to have a health-related context. Studies without an explicitly specified research context are also eligible. '  This seems contradictory.  Why are studies in education topics for example not useful if they are looking at the methods rather than the topic? Just including papers where the topic is not clear seems arbitrary when there may be much to learn from a paper even if it has an engineering topic, say.  Perhaps reword along the lines of 'although studies undertaken in the context of health-related topics are the main focus, studies of citation tracking in other literatures will also be eligible where the focus is on exploring the methods rather than the subject topic'. 
10. Table 1. Publication Type cannot be 'Any' when there are publication types listed as ineligible. The authors should list eligible study types or use some sort of exception wording.
11. Information sources. I am not sure 'free web searching via Google Scholar' is a helpful description, perhaps just say 'searches of Google Scholar' here and further down where it is mentioned again. 
12. 'For citation tracking, we will use Scopus, as this database seems to cover the largest number of relevant citations for the purpose of our review 30. '  There is quite a lot of research that reports that other resources such as Google Scholar or Microsoft Academic provide wider coverage - the authors may wish to consider searching some of the free resources as well (since Scopus alone is not going to find the largest number of results) or finding other reasons for the use of a Scopus only approach. 
13. 'MEDBIB-L' - I think there may be a typo here and it is MEDLIB-L? I think it may also need to be corrected in the Dissemination section. 
14. My understanding of the purpose of a protocol is that it should list what will happen and therefore the resources to be searched should be listed and there should be no examples which imply that other things may be added. So there should be no' e.g.'. 
15. 'text mining approach' - the chapter describes many approaches - which one was used in this case? 
16. 'parts of our textwords' - suggest 'some of our textwords'? 
17. Box 1 - there is harmless redundancy in the search. For example co-citation is searched alone and in combination with other terms - it only needs to be searched alone.  
18. Box 1 - with the focus on searching in the titles, the use of such close adjacency does not seem warranted - it would be more sensitive to use AND for title searching.
19. Box 1 - it might be helpful in the absence of subject headings and the focus on searching mainly in titles to also search the author keywords field.
20. Data charting process: I realise it is difficult to know what will happen but the authors description is not very clear about their plans 'We aim for an iterative data extraction process, but in the final publication, we will provide a detailed overview of extracted data items.'  What is an 'iterative data extraction process' - what might it look like and what does it involve?  
21. Data collection 'preferred taxonomy' - again, I suggest the word you mean is 'terminology' not 'taxonomy'. 
22. ***Data analysis.*** 'To analyse the survey data, we will apply descriptive statistics based on frequencies, percentages, and cross tables.' I think it may be 'cross tabulations' rather than 'cross tables'? 
23. Dissemination of results - it might be useful to also to try to present results at conferences of systematic review experts - Cochrane Colloquium and the Health Technology Assessment International (HTAi) conferences. These organisations also have active information retrieval special interest groups. 
24. Conclusions: 'Depending on the available study landscape' - this seems rather vague to me - it would help the reader if the authors could be more explicit about what they mean. 
25. 'consequently, on the quality of clinical care' - I think it might not only be clinical care that could be impacted - it might be any aspect of health care depending on what is being reviewed e.g. a service, a policy, a new method of organising staff etc.
26. 'in a health-related context may prove relevant also for other academic fields such as social or environmental sciences' - I think that much of what you find may be generalisable to other disciplines, so I suggest that you state this earlier as a possibility.

Is the study design appropriate for the research question?

Yes

Is the rationale for, and objectives of, the study clearly described?

Yes

Are sufficient details of the methods provided to allow replication by others?

Partly

Are the datasets clearly presented in a useable and accessible format?

Not applicable

Reviewer Expertise:

Information retrieval for evidence identification for systematic reviews.

I confirm that I have read this submission and believe that I have an appropriate level of expertise to confirm that it is of an acceptable scientific standard, however I have significant reservations, as outlined above.

## References

[1] : A relevance ranking method for citation-based search results. *Scientometrics*.2017;112(2) : 10.1007/s11192-017-2406-y731-74610.1007/s11192-017-2406-y

[2] : Novel citation-based search method for scientific literature: a validation study. *BMC Medical Research Methodology*.2020;20(1) : 10.1186/s12874-020-0907-510.1186/s12874-020-0907-532028894PMC7006380

# Response

**Published:** 2021-07-09

**Authors:** Julian Hirt

**Affiliations:** University Hospital Basel, Switzerland

Dear Julie Glanville

 We are grateful for your reviewer report and, in particular, for your helpful suggestions for improvement of our scoping review protocol. Below, we provide a detailed point-by-point response with changes that we implemented in version 2 of our manuscript.

 Please note that in response to the comments by reviewer 2 (David Moher), we replaced the planned survey with a planned Delphi study to address the original objective. This change also entails the reformulation of our research questions and the intent to publish the final scoping review and the results of the Delphi study separately.

 We hope our revisions now fully meet your concerns.

 Sincerely yours,

 Julian Hirt, Thomas Nordhausen, Christian Appenzeller-Herzog, and Hannah Ewald

*Reviewer comment: Introduction: 'These references are usually eligible for inclusion into the review and known at the start of the citation search10–12.' I think this could be reworded for clarity along the following lines:*

*'These references are usually eligible for inclusion into the review and some may be known at the beginning of the review and others may emerge as eligible records following the main database searches10–12.'*

*Then perhaps you may not need the sentence that follows the one I am commenting on.*

 Authors’ response: We adapted this sentence and the subsequent one according to your suggestion.

*Reviewer comment: Introduction: 'The taxonomy used to describe the…'. I think it might be better to use a word such as 'terminology' rather than 'taxonomy'.*

 Authors’ response: Thank you. We now use terminology instead of taxonomy throughout the manuscript.

*Reviewer comment: Figure 1. The figure would be more helpful if it could show the relationships of the indirect citation tracking in the Figure 1b perhaps by colour coding. Please check diagrams in the following references which incorporate chronology into the citation picture as well as showing the indirect citation tracking clearly.*

*Belter CW. A relevance ranking method for citation-based search results. Scientometrics. 2017;112(2):731-46.1*

*Janssens A, Gwinn M, Brockman JE, Powell K, Goodman M. Novel citation-based search method for scientific literature: a validation study. BMC Med Res Methodol. 2020;20(1):25.2*

 Authors’ response: We have colour coded the 4 citation tracking techniques as suggested. We had already consulted the citation tracking figures of Belter *et al*. and Janssens. *et al.* amongst others. Furthermore, we have added text to the figure caption. We feel that our figure is now clearer with respect to chronology.

*Reviewer comment: Introduction: 'However, recent guidance suggests that combining several citation tracking methods (e.g. screening cited, citing, co-cited and co-citing references) may be the most effective way to use citation tracking for systematic reviewing.' Please cite the guidance.*

 Authors’ response: We added a reference.

*Reviewer comment: Introduction: ' It rather depends on a variety of factors. For instance, citation tracking may be especially beneficial in case of (i) complex searches (e.g. for reviews on public health topics), (ii) searching for health outcome measurement instruments, or (iii) research areas without consistent taxonomy, with vocabulary overlaps with other fields, or with a lack of index terms in databases (e.g. methodological topics)'. These are all important issues and each deserves a bit more description so that readers can understand the differences. Again I think the word 'taxonomy' should be replaced with 'terminology'.*

 Authors’ response: Thank you for this point! We have revised and elaborated this section.

*Reviewer comment: Introduction: current 'topical reviews' - suggest replacing with 'recent reviews on this topic'. Then please add in more citations so that 'reviews' can be supported with more than one reference.*

 Authors’ response: We adapted the sentence according to your suggestion, thank you.

*Reviewer comment: Introduction: 'health-related systematic literature searching'. This occurs three times and for each occurrence I suggest rewording as follows for clarity: 'literature searching for systematic reviews of health and health-related topics'.*

 Authors’ response: Thank you for your proposition. We now use “systematic literature searching for health-related topics“.

*Reviewer comment: Eligibility criteria. 'evidence retrieval method'. Suggest that 'method' is not needed as the sentence has 'means'.*

 Authors’ response: We deleted the word ‘method’.

*Reviewer comment: 'Eligible studies need to have a health-related context. Studies without an explicitly specified research context are also eligible.' This seems contradictory. Why are studies in education topics for example not useful if they are looking at the methods rather than the topic? Just including papers where the topic is not clear seems arbitrary when there may be much to learn from a paper even if it has an engineering topic, say. Perhaps reword along the lines of 'although studies undertaken in the context of health-related topics are the main focus, studies of citation tracking in other literatures will also be eligible where the focus is on exploring the methods rather than the subject topic'.*

 Authors’ response: We agree that it is interesting to learn about citation tracking from other disciplines. However, we have decided to focus on studies in a health-related context because this enables us to (i) consider the context in which the studies were conducted and to (ii) specifically direct our conclusions and recommendations to the health context. Furthermore, we concentrate on health-related studies for practical reasons facing the high number of references that need screening. We deleted the sentence ‘Studies without an explicitly specified research context are also eligible’ to avoid misunderstandings.

 On that note we also improved our exclusion criteria by adding this criterion: ‘any study only assessing the benefit of combined search methods (whereas the isolated benefit of citation tracking cannot be extracted)’.

*Reviewer comment: Table 1. Publication Type cannot be 'Any' when there are publication types listed as ineligible. The authors should list eligible study types or use some sort of exception wording.*

 Authors’ response: We now use ‘Any reports of empirical studies’.

*Reviewer comment: Information sources. I am not sure 'free web searching via Google Scholar' is a helpful description, perhaps just say 'searches of Google Scholar' here and further down where it is mentioned again.*

 Authors’ response: We now use ‘web searching’ instead of ‘free web searching’ throughout the manuscript.

*Reviewer comment: 'For citation tracking, we will use Scopus, as this database seems to cover the largest number of relevant citations for the purpose of our review30.' There is quite a lot of research that reports that other resources such as Google Scholar or Microsoft Academic provide wider coverage - the authors may wish to consider searching some of the free resources as well (since Scopus alone is not going to find the largest number of results) or finding other reasons for the use of a Scopus only approach.*

 Authors’ response: We agree and now plan forward citation tracking using a triple approach: Scopus, Web of Science, and Google Scholar. We will iteratively repeat citation tracking on newly identified records to include until no further eligible references will be identified. We now describe this new approach in the methods.

*Reviewer comment: 'MEDBIB-L' - I think there may be a typo here and it is MEDLIB-L? I think it may also need to be corrected in the Dissemination section.*

 Authors’ response: The correct name is MEDIBIB-L (the “I” was missing), a mailing list specific to libraries of medicine in the German-speaking area (library = Bibliothek; https://lists.uni-due.de/mailman/listinfo/medbib-l).

*Reviewer comment: My understanding of the purpose of a protocol is that it should list what will happen and therefore the resources to be searched should be listed and there should be no examples which imply that other things may be added. So there should be no' e.g.'.*

 Authors’ response: We deleted ‘e.g.’

*Reviewer comment: 'text mining approach' - the chapter describes many approaches - which one was used in this case?*

 Authors’ response: We used several tools that were used to improve our search. We now list all the tools that we used during search drafting phase in our manuscript and here:

- PubMed PubReMiner: https://hgserver2.amc.nl/cgi-bin/miner/miner2.cgi
- AntConc 3.5.7 (Windows), developed by Laurence Anthony, Faculty of Science and Engineering, Waseda University, Japan
- Yale MeSH Analyzer: http://mesh.med.yale.edu/
- TerMine: http://www.nactem.ac.uk/software/termine/
- Text Analyzer: https://www.online-utility.org/text/analyzer.jsp
- Voyant: https://voyant-tools.org/
- VOSviewer: https://www.vosviewer.comrt

*Reviewer comment: 'parts of our textwords' - suggest 'some of our textwords'?*

 Authors’ response: We agree, thank you.

*Reviewer comment: Box 1 - there is harmless redundancy in the search. For example co-citation is searched alone and in combination with other terms - it only needs to be searched alone.*

 Authors’ response: Yes, these are remnants of a previously more specific search and indeed now redundant. Since we already ran the searches by now, we would prefer to leave the search string exactly as we ran it for precise documentation. However, we noted this in our search files in case of a later update.

*Reviewer comment: Box 1 - with the focus on searching in the titles, the use of such close adjacency does not seem warranted - it would be more sensitive to use AND for title searching.*

 Authors’ response: Again, this is very good input which we noted in our search files in case of a later update. Unfortunately, we already ran the searches by now and are done screening. We are, however, confident that any article that we missed due to the close adjacency will be retrieved through our extensive citation tracking, web searches or expert contacting.

*Reviewer comment: Box 1 - it might be helpful in the absence of subject headings and the focus on searching mainly in titles to also search the author keywords field.*

 Authors’ response: Thank you for the sensible suggestion which we also noted in our search files in case of a later update.

*Reviewer comment: Data charting process: I realise it is difficult to know what will happen but the authors description is not very clear about their plans 'We aim for an iterative data extraction process, but in the final publication, we will provide a detailed overview of extracted data items.' What is an 'iterative data extraction process' - what might it look like and what does it involve?*

 Authors’ response: We revised this paragraph and provide more details: ‘Since we expect heterogeneous studies in terms of aim, design, and methods, we aim for an iterative data extraction process. This allows a flexible and study-specific data extraction process, e.g. by adding previously neglected data extraction items that might contribute to the overall body of knowledge to the data extraction form.’

*Reviewer comment: Data collection 'preferred taxonomy' - again, I suggest the word you mean is 'terminology' not 'taxonomy'.*

 Authors’ response: Indeed, thank you.

*Reviewer comment: Data analysis. 'To analyse the survey data, we will apply descriptive statistics based on frequencies, percentages, and cross tables.' I think it may be 'cross tabulations' rather than 'cross tables'?*

 Authors’ response: We revised this paragraph to address a suitable type of analysis for our data retrieved in Delphi rounds. For free text answers and statements of experts, we will use thematic categorisation. For votes whose results are available or can be converted into numbers, we will use descriptive statistics.

*Reviewer comment: Dissemination of results - it might be useful to also to try to present results at conferences of systematic review experts - Cochrane Colloquium and the Health Technology Assessment International (HTAi) conferences. These organisations also have active information retrieval special interest groups.*

 Authors’ response: Thank you, we added these conferences to our list of potential conferences.

*Reviewer comment: Conclusions: 'Depending on the available study landscape' - this seems rather vague to me - it would help the reader if the authors could be more explicit about what they mean.*

 Authors’ response: We deleted ‘Depending on the available study landscape’ to be more precise.

*Reviewer comment: 'consequently, on the quality of clinical care' - I think it might not only be clinical care that could be impacted - it might be any aspect of health care depending on what is being reviewed e.g. a service, a policy, a new method of organising staff etc.*

 Authors’ response: We agree and revised the sentence to ‘health care’ instead of ‘clinical care’.

*Reviewer comment: 'in a health-related context may prove relevant also for other academic fields such as social or environmental sciences' - I think that much of what you find may be generalisable to other disciplines, so I suggest that you state this earlier as a possibility.*

 Authors’ response: We agree that our results may indeed prove relevant to other disciplines. However, we decided to narrow our research efforts to the health-related fields, so we think this should be rather a part of the discussion/outlook than earlier in the manuscript. We changed the sentence to underline that aspect: “Although we solely focus on a health-related context, it is possible that some of the recommendations developed during this project may prove relevant also for other academic fields such as social or environmental sciences”.
